# Chronic kidney disease is associated with poorer in-hospital outcomes in patients hospitalized with infections: Electronic record analysis from China

**DOI:** 10.1038/s41598-017-11861-2

**Published:** 2017-09-14

**Authors:** Guobin Su, Hong Xu, Gaetano Marrone, Bengt Lindholm, Zehuai Wen, Xusheng Liu, Juan-Jesus Carrero, Cecilia Stålsby Lundborg

**Affiliations:** 1 Global Health – Health Systems and Policy: Medicines, focusing antibiotics, Department of Public Health Sciences, Karolinska Institutet, Stockholm, Sweden; 2 Department of Nephrology, Guangdong Provincial Hospital of Chinese Medicine, The Second Affiliated Hospital, Guangzhou University of Chinese Medicine, Guangzhou City, Guangdong Province China; 3 Division of Renal Medicine and Baxter Novum, Department of Clinical Science, Intervention and Technology, Karolinska Institutet, Stockholm, Sweden; 4 Key Unit of Methodology in Clinical Research (KUMCR), Guangdong Provincial Hospital of Chinese Medicine, The Second Affiliated Hospital, Guangzhou University of Chinese Medicine, Guangzhou City, Guangdong Province China

## Abstract

Predominantly based on studies from high-income countries, reduced estimated glomerular filtration rate (eGFR) has been associated with increased risk of infections and infection-related hospitalizations (IRHs). We here explore in-hospital outcomes of IRHs in patients with different kidney function. A total of 6,283 adults, not on renal replacement therapy, with a discharge diagnosis of infection, and with an eGFR 1–12 months before index hospitalization, were included from four hospitals in China. We compared in-hospital outcomes (death, intensive care unit (ICU) admission, length of hospital stay (LOHS) and medical expenses), between patients with and without chronic kidney disease (CKD, defined as eGFR ≤ 60 ml/min per 1.73 m^2^ of body surface area) by mixed-effects logistic regression model or generalized linear model. The odds for in-hospital mortality (adjusted odds ratios (OR) = 1.41; 95% CI 1.02–1.96) and ICU admission (OR = 2.18; 95% CI 1.64–2.91) were higher among patients with CKD. The median LOHS was significantly higher for CKD patients (11 days vs. 10 days in non-CKD, *P* < 0.001), and inferred costs were 20.0% higher adjusted for inflation rate based on costs in 2012 (*P* < 0.001). Patients with CKD hospitalized with infections are at increased risk of poorer in-hospital outcomes, conveying higher medical costs.

## Introduction

Around 10% of the global adult population has chronic kidney disease (CKD)^1^. In China, the number of patients with CKD is estimated to be 120 million^2, 3^. Infection is an important cause of mortality and hospitalization in patients with CKD, contributing to a considerable health care resource burden^4^.

Predominantly based on studies from high-income countries, an increased risk of infections has been observed in individuals with CKD^5–7^. CKD has also been suggested to be a risk factor for infection-related hospitalizations (IRHs) as well as for pneumonia and sepsis-related mortality^8–17^.

Allocation of resources should be prioritized to those with higher risks of poorer outcomes as stressed in China’s recent health care reform^18, 19^. If CKD is a marker for poorer in-hospital outcomes of IRHs, this would be useful for risk stratification, clinical management, as well as for allocation of health care resources at the societal level. It would also have implications for the so-called single disease reimbursement policy in China healthcare reform^18^. Under this policy, hospitals are responsible for all medical expenses during patients’ stay in hospitals. Patients pay for a certain proportion of the total medical expense when they are discharged. For the rest of the medical expenses during patients’ stay, hospitals will get the same amount of reimbursement from the health insurance run by the government, if the patients are admitted for the same reason, regardless of their comorbidities. However, it is unclear in higher middle-income countries, such as China, whether and to what extent CKD is associated with in-hospital outcomes that utilize more healthcare resources compared with patients without CKD. These may include, for example, a greater likelihood of intensive care unit (ICU) admission, longer lengths of hospital stay and higher medical expenses.

The aim of this study was to characterize the pattern of infections in hospitalized CKD patients and quantify hospital related outcomes (in-hospital mortality, ICU admission frequency, the length of hospital stay) as well as medical expenses during hospitalization, compared with non-CKD patients.

## Results

**Figure 1:**
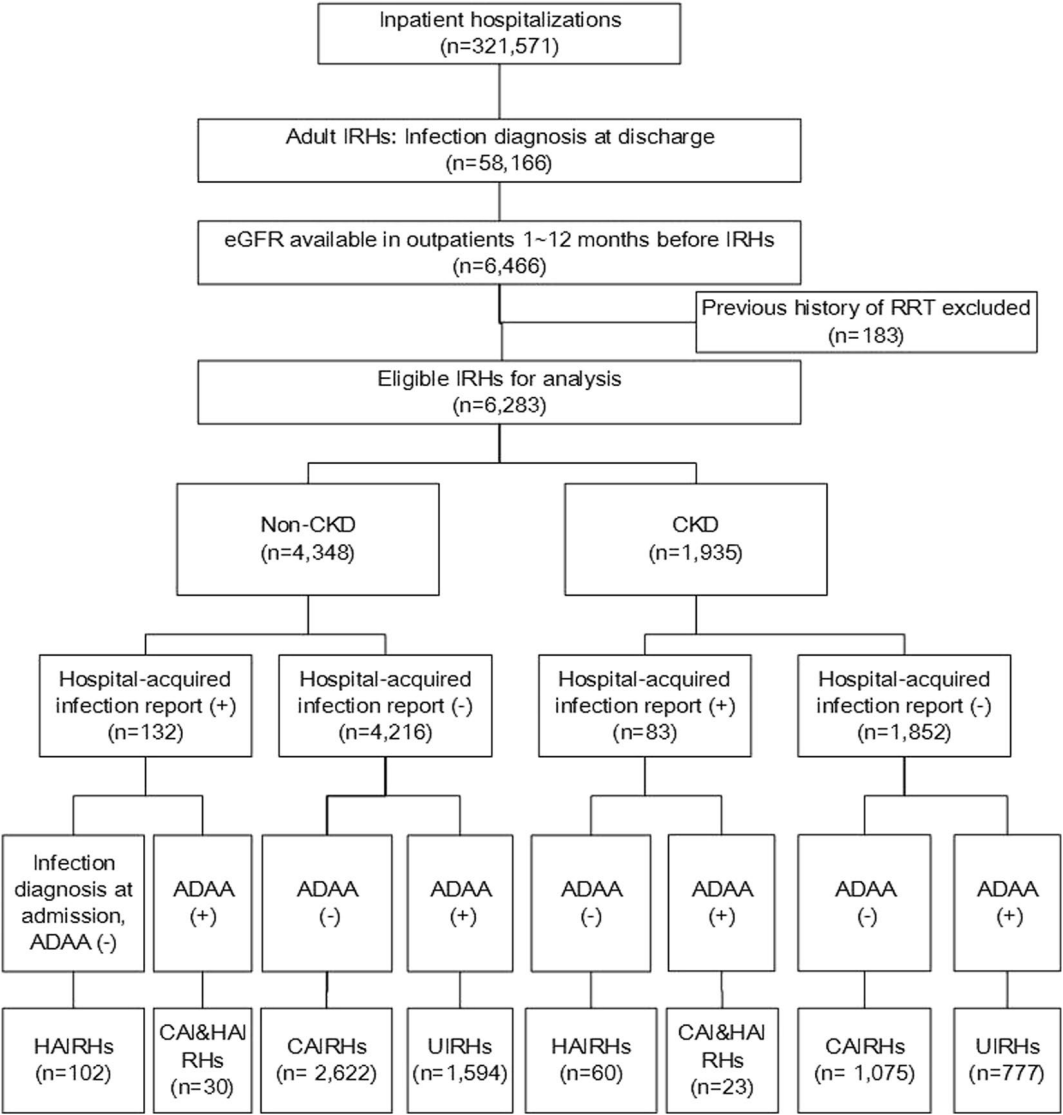
Flowchart of types and eligible infection-related hospitalizations for analysis. Abbreviations: IRHs, infection-related hospitalizations; RRT, renal replacement therapy; CAIRHs, community-acquired infection-related hospitalizations; HAIRHs, health care-associated IRHs; CAI&HAIRHs, both community-acquired and health care-associated IRHs; UIRHs, undefined infection-related hospitalizations.

We identified 321,571 inpatient hospitalizations. Among them, 58,166 were considered adult IRHs, accounting for 18.1(58,166/321,571)% of the total inpatient hospitalization. For the IRHs, only 11.1% (6,466/58,166) had an eligible serum creatinine to estimate kidney function. We excluded 183 individuals undergoing RRT. The study, therefore, analyzed data from 6,283 IRHs (Fig. 1).

At the time of discharge, 83% (5,214/6,283) of all IRHs had one infection diagnosis, 13.8% (869/6,283) had two infection diagnoses, and 3.2% (200/6,283) had three or more infection diagnoses.

### Baseline demographics of patients with and without CKD

Patients with CKD were older, more likely to be male, and had more comorbidities, compared with patients without CKD (Table 1). There were no differences in the proportion of surgrical procedures during the hospitalization between the two groups.

**Table 1:** Baseline 6,283 patient demographics and types of infection-related hospitalizations stratified by the presence/absence of CKD in Guangzhou, China.

	Total (n = 6,283)	Non-CKD (n = 4,348)	CKD (n = 1,935)	*P value
Age (y, median) [interquartile range]	63 [49–77]	66 [53–77]	77 [68–83]	<0.001
Female, n (%)	3,122 (49.7)	2,212 (50.1)	910 (47.0)	0.005
Procedure or surgery during hospitalization, n (%)	1,896 (30.1)	1,317 (30.3)	579 (29.9)	0.77
Charlson comorbidities index, median [interquartile range]	1 [0–2]	1 [0–2]	2 [1–3]	<0.001
*Single Comorbidities*				
Acute myocardial infarction, n (%)	116 (1.9)	43 (1.0)	73 (3.8)	<0.001
Congestive heart failure, n (%)	500 (8.0)	236 (5.4)	264 (13.6)	<0.001
Peripheral vascular disease, n (%)	105 (1.7)	39 (0.9)	66 (3.4)	<0.001
Cerebral vascular accident, n (%)	1,740 (27.7)	988 (22.7)	752 (38.9)	<0.000
Dementia, n (%)	47 (0.8)	21 (0.5)	26 (1.3)	<0.001
Chronic pulmonary disease, n (%)	1,741 (27.7)	1,230 (28.3)	511 (26.4)	0.12
Connective tissue disorder, n (%)	205 (3.3)	140 (3.2)	65 (3.4)	0.77
Peptic ulcer, n (%)	223 (3.6)	139 (3.2)	84 (4.3)	0.02
Liver disease, n (%)	157 (2.5)	115 (2.6)	42 (2.2)	0.26
Diabetes, n (%)	1,627 (25.9)	960 (22.1)	667 (34.5)	<0.001
Diabetes complications, n (%)	355 (5.7)	140 (3.2)	215 (11.11)	<0.001
Paraplegia, n (%)	2 (0.03)	2 (0.05)	0 (0)	0.34
Cancer, n (%)	1,048 (16.7)	795 (18.3)	253 (13.1)	<0.001
Metastatic cancer, n (%)	24 (0.4)	20 (0.5)	4 (0.2)	0.13
Severe liver disease, n (%)	54 (0.9)	37 (0.9)	17 (0.9)	0.9
AIDS, n (%)	0 (0)	0 (0)	0 (0)	1
*Types of IRH*				0.01
Community-acquired IRHs, n (%)	3,697 (58.8)	2,622 (60.3)	1,075 (55.6)	<0.001
Hospital-acquired IRHs, n (%)	162 (2.6)	102 (2.4)	60 (3.1)	0.081
CAI&HAIRHs, n (%)	53 (0.8)	30 (0.7)	23 (1.2)	0.05
Undefined IRHs, n (%)	2,371 (37.7)	1,594 (36.7)	777 (40.2)	0.008

*t-test or Analysis of variance or Chi^2^ test, or Wilcoxon rank test if not normal distribution.

Abbreviations: AIDS, acquired immunodeficiency syndrome; IRH: infection-related hospitalization; CAIRHs: community-acquired infection-related hospitalizations; HAIRHs: health-care associated infection-related hospitalizations.

### Types of IRHs and spectrum of infections

**Figure 2:**
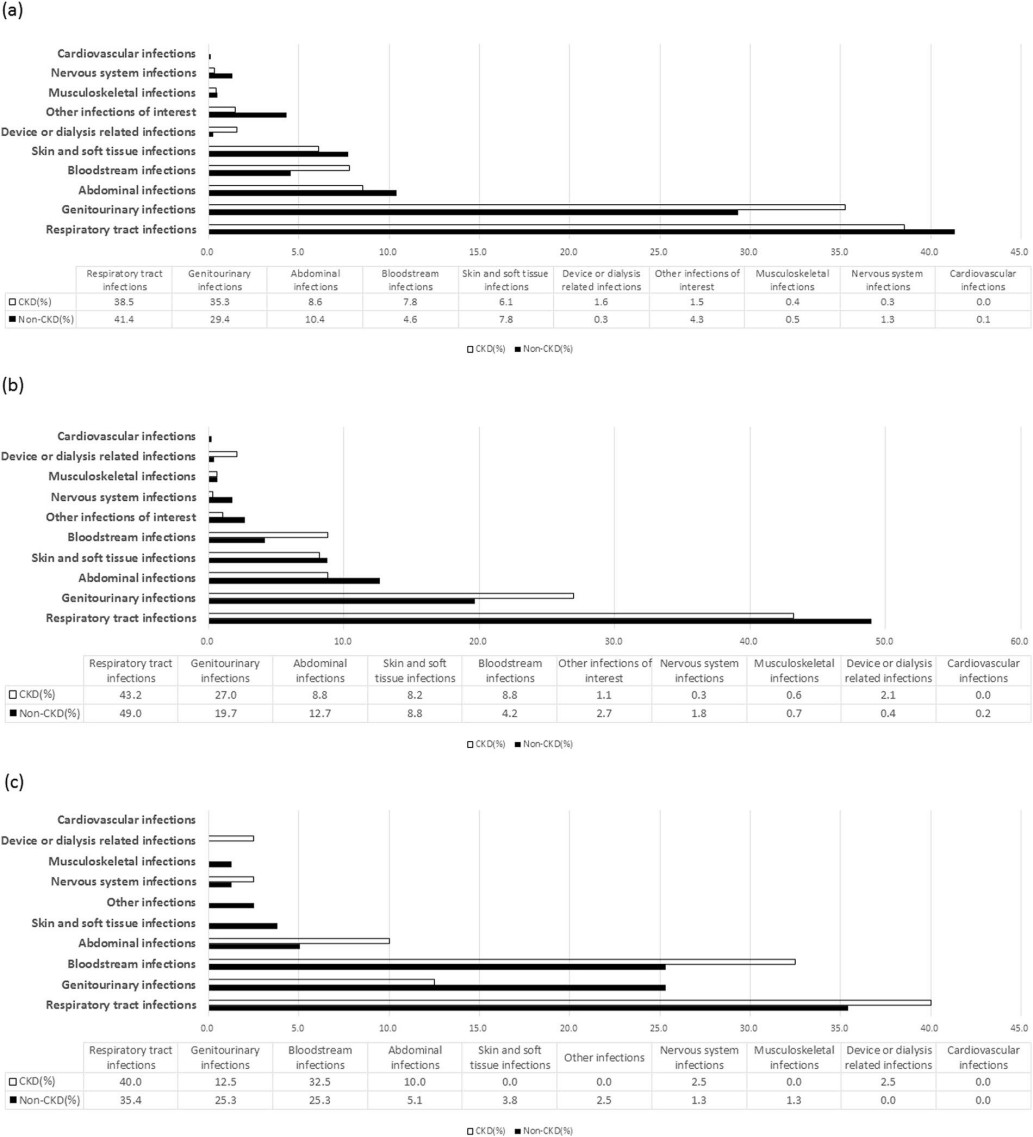
The spectrum of infections in different types of infection-related hospitalizations (IRHs) in 6,283 patients with/without CKD in Guangzhou, China. (**a**) type-specific infections in overall IRHs; (**b**) type-specific infections in community-acquired IRHs (CAIRHs); (**c**) type-specific infections in health-care-associated IRHs (HAIRHs).

The majority of IHRs were classified as being community-acquired (n = 3,697, 58.8%), followed by undefined (n = 2,371, 37.7%), healthcare-acquired (n = 162, 2.6%), and IRHs with both CAIRHs and HAIRHs (n = 53, 0.8%). The pattern of IRH types did not seem to differ between the groups (Table 1). The pattern of infections was also similar between groups, with respiratory tract infections, genitourinary tract and abdominal infections as the top-three causes, followed by skin/soft tissue, and bloodstream infections (Fig. 2).

### In-hospital mortality

**Figure 3:**
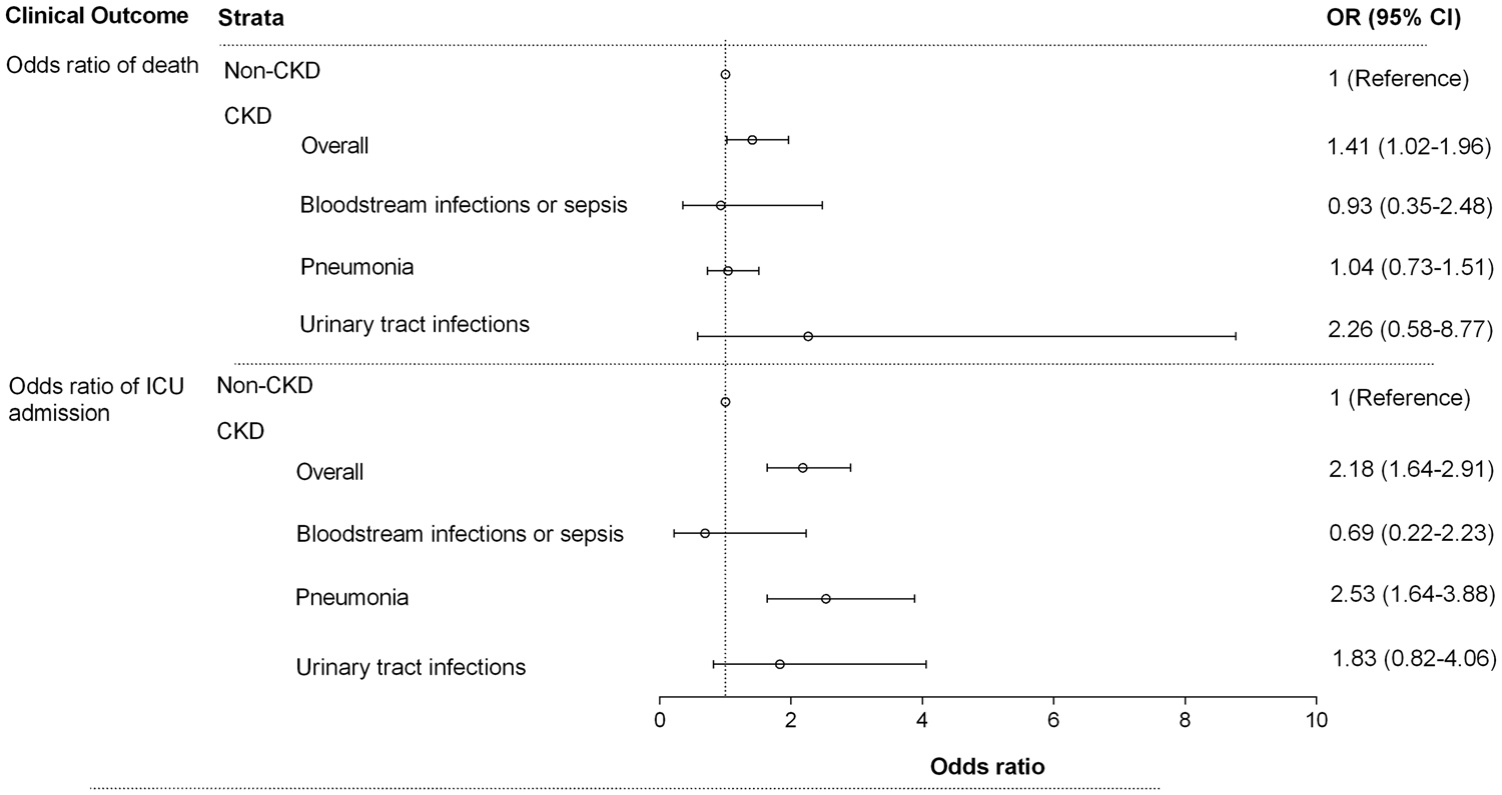
Odds Ratio of mortality and intensive care unit admission in 6,283 patients with/without CKD in Guangzhou, China. CKD was defined as eGFR < 60 ml/min per 1.73 m^2^ of body surface area, while Non-CKD was defined as eGFR ≥ 60 ml/min per 1.73 m^2^ of body surface area, regardless of concomitant albuminuria. Odds ratio adjusted for age, sex and Charlson comorbidity index.

A total of 166 (3.8%) non-CKD patients died in-hospital, while 115 (5.9%) died in the CKD group **(**Table 2 **)**. The odds of in-hospital mortality were higher among patients with CKD compared with non-CKD patients (odds ratio, OR = 1.41; 95% CI 1.02–1.96 in the fully adjusted model). In subgroup and sensitivity analysis, ORs for in-hospital mortality were higher (but not statistically significant) among patients with CKD compared with non-CKD patients in CAIRHs (OR = 1.34; 95% CI 0.86–2.09), pneumonia (OR = 1.04; 95% CI 0.73–1.51), UTIs (OR = 2.26; 95% CI 0.58–8.77) and in those excluding readmission (OR = 1.18; 95% CI 0.84–1.96), (Fig. 3 & Supplement Tables 2 & 4 **)**.

**Table 2:** Odds Ratios (OR) and 95% confidence intervals for in-hospital death in 6,283 patients with/without CKD in Guangzhou, China.

In-hospital deaths	Total (n = 6,283)	Non-CKD (n = 4,348)	CKD (n = 1,935)	P-value
Overall, n (%)	281 (4.3)	166 (3.8)	115 (5.9)	<0.001*
Crude OR		1 (Ref)	1.59 (1.24–2.04)	<0.001
Adjusted OR (Model 1)		1 (Ref)	1.32 (1.01–1.71)	0.04
Adjusted OR (Model 2)		1 (Ref)	1.41 (1.02–1.96)	0.04
CAIRHs, n (%)	148 (4.0)	87 (3.3)	61 (5.7)	<0.001*
Crude OR		1 (Ref)	1.75 (1.25–2.45)	<0.001
Adjusted OR (Model 1)		1 (Ref)	1.43 (0.97–2.11)	0.06
Adjusted OR (Model 2)		1 (Ref)	1.34 (0.86–2.09)	0.20
HAIRHs, n (%)	15 (9.3)	9 (8.8)	6 (10.0)	0.80*
Crude OR		1 (Ref)	1.14 (0.39–3.41)	0.80
Adjusted OR (Model 1)		1 (Ref)	0.89 (0.28–2.82)	0.26
Adjusted OR (Model 2)		1 (Ref)	0.87 (0.27–2.70)	0.79

Model 1 Adjusted for Age and Sex.

Model 2 Adjusted for Age, sex and Charlson comorbidity index.

*Chi^2^ test.

Abbreviations: CAIRHs: community-acquired infection-related hospitalizations; HAIRHs: health-care associated infection-related.

### ICU admission during the hospital stay

The ICU admission rates were 12.2% and 5.5% in patients with and without CKD, respectively. The odds of ICU admission were higher among patients with CKD (OR = 2.18; 95% CI 1.64–2.91 in the fully adjusted model). A similar pattern was observed for both CAIRHs (OR = 2.01; 95% CI 1.34–3.02) and HAIRHs (OR = 2.48; CI 1.08–5.70) (Table 3). We further analyzed single infection categories, and found that the increased odds for ICU admission in patients with CKD remained statistically significant in the fully adjusted model for pneumonia (OR = 2.53; 95% CI 1.64–3.88) in CKD patients (Fig. 3 & Supplement Table 3). In sensitivity analysis, the higher odds for ICU admission remained significant in those, after excluding readmitted patients (Supplement Table 4),

**Table 3:** Odds Ratios (OR) and 95% confidence intervals for intensive care unit admission in 6,283 patients with/without CKD in Guangzhou, China.

Intensive care unit admission	Total (n = 6,283)	Non-CKD (n = 4,348)	CKD (n = 1,935)	*P-value
Overall, n (%)	474 (7.5)	239 (5.5)	235 (12.2)	<0.001
Crude OR		1 (Ref)	2.37 (1.97–2.87)	<0.001
Adjusted OR (Model 1)		1 (Ref)	2.32 (1.75–3.09)	<0.001
Adjusted OR (Model 2)		1 (Ref)	2.18 (1.64–2.91)	<0.001
CAIRHs, n (%)	263 (7.1)	141 (5.4)	122 (11.4)	<0.001
Crude OR		1 (Ref)	2.25 (1.74–2.91)	<0.001
Adjusted OR (Model 1)		1 (Ref)	2.12 (1.41–3.16)	<0.001
Adjusted OR (Model 2)		1 (Ref)	2.01 (1.34–3.02)	0.001
HAIRHs, n (%)	33 (20.37)	14 (13.7)	19 (31.7)	0.006
Crude OR		1 (Ref)	2.91 (1.30–6.51)	0.006
Adjusted OR (Model 1)		1 (Ref)	2.48 (1.08–5.68)	0.03
Adjusted OR (Model 2)		1 (Ref)	2.48 (1.08–5.70)	0.03

Model 1 Adjusted for Age and Sex.

Model 2 Adjusted for Age, sex and Charlson comorbidity index.

*Chi^2^ test.

Abbreviations: CAIRHs: community-acquired infection-related hospitalizations; HAIRHs: health-care associated infection-related hospitalizations.

### Days of hospital stay and medical expense

The median length of stay for CKD patients was 11 [8–15] days, which was significantly longer (p < 0.001) than the length of stay for non-CKD patients, 10 [7–14] days. In community-acquired IRHs and in patients who survived to discharge, the median length of stay for CKD patients was also longer than that for patients without CKD (Supplemental Tables 5 & 6 **)**. Compared with non-CKD patients, medical expenses in patients with CKD were higher by a median of 2400 RMB (around 350 USD) per hospitalization, corresponding to 20.0% increase in total medical expenses adjusted for inflation rate based on costs in 2012 (Table 4). In community-acquired IRHs and in patients who survived to discharge, the median total medical expenses for CKD patients was also higher than that for patients without CKD (Supplemental Tables 5 & 6 **)**.

**Table 4:** The length of hospital stay and associated medical expenses in 6,283 patients with/without CKD in Guangzhou, China.

	Non-CKD	CKD	Difference in (%)	**P-value	***Difference adjusted by age and sex
Median days of hospital stay[IQR]	10 [7–14]	11 [8–15]		<0.001	
Total medical expenses (Median, RMB)	11966.45	14363.65	20.0	<0.001	1501.7
Provided medicines (antibiotics excluded)	4024.61	5275.92	31.1	<0.001	401.1
Antibiotics	697.1	1095.41	57.1	<0.001	74.8
Ward-related	1522.29	2027.87	33.2	<0.001	320.6
Investigation-related	2552.24	2888.19	13.2	<0.001	444.1
Non-surgical therapies	1953.27	2750.04	40.8	<0.001	396.0
****Surgical therapies, n, (IQR, RMB)	0 (0–253.37)	0 (0–224.04)		0.1	

Data from electronic medical record database in Guangdong provincial hospital of Chinese medicine.

Ward-related costs consider all other cost incurred while in the ward.

Investigation-related costs consider pathology, laboratory, imaging and consumable items.

The cost of non-surgical therapies includes physiotherapy, acupuncture, injection, etc.

Cost of surgical therapies include those of anesthesia and related materials.

Difference = [(Cost in CKD–Cost in Non-CKD)/Cost in Non-CKD] × 100%.

All the medical costs were adjusted for inflation rate based on cost in 2012; Inflation rate equals to 2.62% for 2013, 1.92% for 2014 and 1.44% for 2015 from http://www.inflation.eu/inflation-rates/china/historic-inflation/cpi-inflation-china.aspx

**Wilcoxon rank test.

***General linear model adjusted for age and sex.

****Not every patient had a surgery.

Abbreviations: IQR: interquartile range.

## Discussion

Our study is the first to quantify the spectrum of infections and associated in-hospital outcomes of IRH in patients with and without CKD in China. We report that CKD patients hospitalized with infections have increased rates of deaths and ICU admissions, resulting in higher health care resource consumption (lengths of hospital stay and total medical expenses), compared with patients with normal renal function. Our study builds on previous findings that CKD is a risk factor for poor outcomes of IRHs in terms of more frequent admissions to ICU, longer hospital stays, and higher medical costs, in addition to higher mortality.

Increased mortality of IRHs has previously been observed in patients with CKD^8–16^. Fried *et al*. reported that patients with CKD had a 2-fold greater risk of infection-related mortality in the Cardiovascular Health Study^15^. This association was further confirmed in a study from NHANES III^10^ as well as a study in Hong Kong (which only included patients age above 65 years old)^11^. For other specific infections, previous studies have reported that CKD was associated with increased 28-day and 1-year mortality in septic shock^16^, 90-day mortality in sepsis^13^, 30-day mortality in bloodstream infections^8^, and 30-day mortality in pneumonia^9, 14^. In addition to mortality, we included ICU admission rate and length of hospital stay as clinical outcomes in our study. We identified that the risk of ICU admission and longer hospital stay was higher among patients with CKD as compared to those without CKD.

A number of reasons may explain the associations between CKD and poor clinical outcomes related to infections. We assumed that some factors associated with CKD patients would account for higher in-hospital mortality; ICU admission rate and longer hospital stay in patients with CKD, such as older age, and more comorbidities. However, the association persisted after controlling for these factors, suggesting that the associations are not explained entirely by these factors. Other factors might contribute to the poor outcome in CKD, such as poorer responses to treatment, immune dysfunction with increased susceptibility to infections, and a higher incidence of dehydration that may induce acute kidney injury during infection^4, 20–24^. Besides, a higher rate of infections and greater exposure to antibiotics might lead to a higher risk of getting infections caused by antibiotic resistant pathogens in patients with CKD, resulting in higher mortality. The association between CKD and antibiotic-resistant bacteria has been suggested in a previous study^25^, while excess mortality and length of hospital stay (LOS) associated with antibiotic resistance has been shown earlier^26^. Last but not least, different patterns of infections might result in different in-hospital mortality and ICU admission rates. But, the patterns of infections types did not differ between patients with and without CKD, and the associations of infections with poorer outcomes remained after controlling for types of infections.

In a sensitivity analysis, in-hospital mortality and ICU admission remained higher in patients with CKD for most infection types, but not statistically significantly. This trend is in line with previous studies^8, 9, 14^. In general, the lack of statistical significance might be related to the smaller sample size when we categorize cause-specific outcomes. Regarding patients with bloodstream infections or sepsis, in-hospital mortality and ICU admission was lower in patients with CKD. This may be related to the small numbers of events due to high mortality in sepsis.

Regarding economic burden, the total medical expenses of IRHs was higher in those with CKD than in those without. Longer hospital stays might partly explain the higher medical costs. Increased severity of infection in CKD, indicated by poorer clinical outcomes, might also contribute to the higher medical costs. Besides, the types of antibiotics in patients with CKD might be more expensive than in those without, as antibiotics used in patients with CKD need to be selected to have less nephrotoxicity. According to the single disease reimbursement payment promoted in the China’s healthcare reform, the reimbursement to the hospital is the same if patients are admitted with the same illness, regardless of their coexisting comorbidities^18, 19^. The findings of our study indicated that patients hospitalized with infections have higher medical expenses if they also have CKD, which could justify adjustments of reimbursements under the single disease reimbursement payment policy.

Our study has several strengths: we comprehensively examined the spectrum of infections in IRHs, both community-acquired and hospital-acquired infections, identifying potential areas for which targeted interventions may be helpful. Many previous studies analyzed registry data with large sample sizes but lack detailed information about clinical outcomes and economic burdens of IRHs. We were able to examine associations between CKD and in-hospital a range of in-hospital outcomes, furthering our understanding of how certain characteristics predispose to in-hospital death, ICU admission, longer hospital stays and higher medical costs in China.

There are limitations to our study that should be taken into consideration when interpreting the results: 1) We only included those with at least one serum creatinine record in outpatient care between one and twelve months before IRHs, in only 11% of all eligible patients. 2) We only used the closest serum creatinine 1–12 months before hospitalization as a proxy of the kidney function, as we expected the kidney function to be relatively stable over one year. From previous studies in Chinese patients with stage 4 CKD, the median rate of GFR decrease was around 6.8 ml/min per 1.73 m^2^ of body surface area per year^27^. Misclassification might, however, exist due to any change of kidney function during this period. 3) We might have overestimated the proportion of patients with reduced eGFR hospitalized with infections due to only using a single GFR estimate^28^. Only very few patients had at least one record of albumin-creatinine ratio or protein creatinine ratio in outpatient care. Thus, we could not further analyze the role of proteinuria. 4) Despite adjustment for some relevant covariates, we cannot eliminate the potential for residual confounding factors, such as smoking status, alcohol consumption, inability to ambulate or transfer, which were not available in our database. However, given the magnitude of the odds ratios for participants with CKD, it is unlikely that further adjustment for these covariates would negate the observed associations. 5) We did not have a regional hospitalization registry that would have allowed us to identify previous admissions and patients hospitalized with infection in GDHCM and referred to other hospitals before recovery. In this case, we could not follow up their outcomes (in-hospital death, ICU admission, or length of hospital stay to get recovered). However, the proportion of patients referred to other hospitals before recovery is minimal as GDHCM is one of the main referral hospitals in this area. We might have misclassified the CAIRHs and HAIRHs: for example, patients who had healthcare-associated pneumonia, discharged from other hospitals, readmitted to GDHCM for pneumonia within three days, would be misclassified as CAIRHs, not HAIRHs. This would, however, not affect the association between CKD and clinical outcomes.

Our results may not be generalizable to all types of IRHs. We excluded specific types of infection and we included a limited set of organ systems/sites of infection. These have covered all common acute infections. The list has been used in a previous study, which might be useful for future comparison^29^. Besides, all infections in any position of discharge diagnosis would be considered as IRHs. Thus, the infection might not be the primary reason for hospitalization, and we might therefore overestimate the prevalence of hospitalization due to infections.

In conclusion, our findings highlight the poorer clinical outcomes and higher health-care resource consumption of hospitalizations for patients with infections who also have chronic kidney disease. Of note, the observed difference between CKD and non-CKD in the present study could, however, be changed in the long term and in a different context. For patients with CKD, infection prevention strategies should focus on respiratory tract infections and genitourinary tract infections. These patients need to be carefully monitored to prevent modifiable adverse outcomes.

## Methods

### Study Design

This was an observational study using electronic health records from four hospitals.

### Setting and data source

We used data from Guangdong Provincial Hospital of Chinese Medicine (GDHCM). GDHCH is located in Guangzhou city with a population of 13,501,100 residents as of 2015^30^. GDHCM has four hospital branches located in different districts of Guangzhou city, and serves as one of the main referral centers for these districts, with over five million outpatient visits and 70,000 inpatients per year. The four hospitals share the same electronic medical record database (EMRD), developed by International Business Machines Corporation (IBM), which includes all inpatient and outpatient medical records as well as costs invoiced.

### Study population

Inclusion criteria were: adult (>18 years) hospitalized patients with any *International Classification of Diseases, Tenth Revision, Clinical Modification (ICD-10-CM)* discharge diagnosis of infections (infection-related hospitalizations, IRHs), between August, 2012 and December, 2015, and presence of at least one outpatient serum creatinine (sCr) measurement between one and twelve months before hospitalization. The reason for this timeframe was to have a more reliable estimation of renal function from serum creatinine tests not influenced by the acute nature of the hospitalization. The only exclusion criterion was if patients had been undergoing renal replacement therapy (RRT, kidney transplantation or dialysis). Since we included only those with serum creatinine at the outpatient visit, no one was lost to follow-up at the time of discharge from GDHCM. The ethics board committee of GDHCM provided ethical approval for the study (B2016–194–01). Informed consent was not needed since the study only involved analysis of anonymized existing data and records. The study was carried out in accordance with Declaration of Helsinki, International Ethical Guideline for Biomedical Research Involving Human Subjects.

### Selection of infection diagnostic codes

We limited our examination to the infection-related diagnoses outlined in the supplemental material^29^ (Supplement Table 1). The following discharge diagnoses were not considered in this study: those commonly found in only infants or children, pregnancy-related infections, delivery-related infections, oral/mouth infections, ear infections, eye infections, pancreatitis, thyroiditis, pituitary gland, infections specified as chronic, chronic hepatitis B and C virus, HIV (human immunodeficiency virus), cholecystitis associated with cholelithiasis/choledocholithiasis, sexually transmitted infections, and parasitic or protozoal diseases.

The selected infections were classified broadly into mutually exclusive categories:

1. *Respiratory tract infection, including pneumonia;*
2. *Genitourinary infections, including urinary tract infections;*
3. *Bloodstream infections or sepsis;*
4. *Abdominal infections*
5. *Skin and soft tissue infections;*
6. *Cardiovascular infections;*
7. *Musculoskeletal infections;*
8. *Nervous system infections;*
9. *Device or dialysis related infections;*
10. *Other infections of interest*.

### Types of infection-related hospitalizations

IRHs were sub-classified as health care-associated IRH (HAIRHs), community-acquired IRH (CAIRHs), both community-acquired and health care-associated IRH (CAI&HAIRHs) and undefined IRH (UIRHs). HAIRHs were defined as the onset of infection after 48 hours after admission^31^ and confirmed by health care-associated infection report in the database which was audited by professionals of infections in GDHCM. CAIRHs were defined as those with infection diagnosis at admission. We considered CAI&HAIRHs as those that had both an infection diagnosis at admission and a health care-associated infection report. UIRHs were those that did not fulfill any of the criteria above (Fig. 1).

### Renal function estimation

eGFR was estimated by sCr concentration, age, and gender according to the established Kidney Disease Improving Global Outcome initiative, and calculated by the CKD-EPI (CKD Epidemiology Collaboration) formula^32^. We first identified patients with infection-related hospitalization and traced them back to find those with a serum creatinine value at an outpatient visit. If more than one value was available, we chose the eGFR value closest in time to the hospitalization (1–12 months before) as a proxy of the kidney function. The reason for this was to have an estimation of renal function from serum creatinine tests not influenced by the acute nature of the hospitalization. CKD was defined as eGFR < 60 ml/min per 1.73 m^2^ of body surface area, while non-CKD was defined as eGFR ≥ 60 ml/min per 1.73 m^2^ of body surface area, regardless of concomitant albuminuria.

### Outcomes

Outcomes data extracted for analysis included: in-hospital mortality, admission to an intensive care unit (ICU), the length of hospital stay and medical expenses. Medical costs during hospitalization were extracted from the billing system in GDHCM. Total medical costs included medicine costs, ward-related costs (considering all other cost incurred while in the ward), investigation-related costs (considering pathology, laboratory, imaging and consumable items), costs of non-surgical therapies (physiotherapy, acupuncture, injection, etc.) and costs of surgical therapies (including anesthesia and related materials).

### Covariates

Age, sex, and data on surgical procedures during hospitalization were obtained from the EMRD in GDHCM. Comorbid conditions considered the classification of Charlson comorbidities index using established *ICD-10* algorithms, including acute myocardial infarction, congestive heart failure, peripheral vascular disease, cerebral vascular accident, dementia, chronic pulmonary disease, connective tissue disorder, peptic ulcer, liver disease, diabetes, diabetes complications, paraplegia, cancer, metastatic cancer, severe liver disease, acquired immunodeficiency syndrome (AIDS)^33^.

### Statistical analysis

All information relating to study outcomes (in-hospital death, ICU admission, the length of hospital stay and medical costs) and covariates (age, sex, comorbidities) in our population were automatically generated from the electronic medical record system. All the data in our study were complete, with no missing data. Numerical variables were summarized using mean ± standard deviation, or median and interquartile range, as appropriate, while categorical variables were summarized using proportions. Differences regarding baseline characteristics, the length of hospital stay, admission to ICU, mortality and medical expenses between patients with and without CKD were compared using ANOVA/ Kruskal-Wallis tests, t-test/Mann-Whitney U test, or chi-square or Fisher´s exact test, as appropriate. Considering the question if in-hospital death would influence the length of hospital stay and medical cost, we did subgroup analysis in patients who survived or died during hospitalization. Given inflation rate changed overtime, we compared medical expenses between those with and without CKD adjusted for inflation rate based on costs in 2012.

Regarding multiple episodes, we used a mixed-effects logistic regression model to set multiple stays within a patient as a cluster and patients as a random effect, to calculate the odds ratio (OR) of death and ICU admission during hospitalization. Model 1 controlled for the potential confounding effects of age and sex. Model 2 controlled for age, sex and Charlson comorbidity index (excluding renal disease score). Further, we repeated the logistic analysis to calculate the odds ratio (OR) of death and ICU admission in patients excluding those with readmissions. To examine factors associated with the length of hospital stay and estimation of total medical cost, we used generalized linear regression models controlling for age and sex. For this analysis we did not adjust for the cluster effect within patients since we considered any hospital stay as an independent statistical unit. A P-value < 0.05 was considered significant. Additionally, we repeated the primary analysis for cause-specific infection-related outcomes including blood stream infections or sepsis, pneumonia and urinary tract infections. All statistical analyses were performed using STATA version 14.2 (StataCorp, College Station, TX, USA).

### Data availability statement

The datasets generated during and/or analysed during the current study are available from the corresponding author on reasonable request and with permission of Guangdong provincial hospital of Chinese medicine.

### Third party right

The images/drawings/photographs in this study were created by the authors of this paper, not from a third party.

## Electronic supplementary material

Supplement
