# Supplementary material for: Chronic kidney disease is associated with poorer in-hospital outcomes in patients hospitalized with infections: Electronic record analysis from China
Source: Sci Rep. 2017 Sep 14;7:11530. doi: 10.1038/s41598-017-11861-2 (PMC5599500; doi:10.1038/s41598-017-11861-2)
Supplement: Supplementary file 1 — Supplement [file 41598_2017_11861_MOESM1_ESM.pdf]

Chronic kidney disease is associated with poorer in-hospital outcomes in patients hospitalized with infections: Electronic record analysis from China

Guobin Su[1,2], Hong Xu[3], Gaetano Marrone[1], Bengt Lindholm[3], Zehuai Wen[4], Xusheng Liu[2], Juan-Jesus Carrero[3], Cecilia Stålsby Lundborg[1]

[1]Global Health – Health Systems and Policy: Medicines, focusing antibiotics, Department of Public Health Sciences, Karolinska Institutet, Stockholm, Sweden;

[2]Department of Nephrology, Guangdong Provincial Hospital of Chinese Medicine, The Second Affiliated Hospital, Guangzhou University of Chinese Medicine, Guangzhou City, Guangdong Province, China;

[3]Division of Renal Medicine and Baxter Novum, Department of Clinical Science, Intervention and Technology, Karolinska Institutet, Stockholm, Sweden;

[4]Key Unit of Methodology in Clinical Research (KUMCR), Guangdong Provincial Hospital of Chinese Medicine, The Second Affiliated Hospital, Guangzhou University of Chinese Medicine, Guangzhou City, Guangdong Province, China.

Corresponding author:

Xusheng Liu

Department of Nephrology, Guangdong Provincial Hospital of Chinese Medicine, The Second Affiliated Hospital, Guangzhou University of Chinese Medicine, Guangzhou City, Guangdong Province, China;

Tel:+86-81887233-34330 Fax: +86-81887233-34330

Email: xushengliu801@126.com or liuxusheng@gzucm.edu.cn

Supplement Table 1. ICD-10 codes for all and different types of infections

Infection categories	ICD-10 codes
Respiratory tract infections	
Non-pneumonia respiratory tract infections	A06.5 A20.2 A21.2 A36.0 A36.1 A36.2 A36.8 A36.9 A37.0 A37.1 A37.8 A37.9 A38.x B00.2 B08.5 B33.4 B44.2 B48.1 J10.8 J00.x J01.0 J01.1 J01.2 J01.3 J01.4 J01.8 J01.9 J02.0 J02.8 J02.9 J03.0 J03.8 J03.9 J04.0 J04.1 J04.2 J05.0 J05.1 J06.0 J06.8 J06.9 J09.x J10.1 J11.1 J11.8 J20.0 J20.1 J20.2 J20.3 J20.4 J20.5 J20.6 J20.7 J20.8 J20.9 J21.0 J21.8 J21.9 J22.x J34.0 J39.0 J39.1 J40.x J41.1 J44.0 J47.x J85.2 J85.3 J86.0 J86.9 J38.3 J38.7 J39.2 J39.8 J95.0 J98.5 J98.9
Pneumonia	A22.1 A31.0 A42.0 A43.0 A48.1 B01.2 B05.2 B25.0 B37.1 B38.0 B38.2 B39.2 B40.2 B41.0 B42.0 B44.0 B44.1 B45.0 B46.0 J10.0 J11.0 J12.0 J12.1 J12.2 J12.8 J12.9 J13.x J14.x J15.0 J15.1 J15.2 J15.3 J15.4 J15.5 J15.6 J15.7 J15.8 J15.9 J16.0 J16.8 J18.0 J18.1 J18.2 J18.8 J18.9 J85.0 J85.1 J98.4
Genitourinary infections	
Urinary tract infections (UTIs)	N10.x N12.x N13.6 N15.1 N30.0 N30.3 N34.0 N34.1 N34.2 N34.3 N39.0 N15.9 N28.8 N30.8 N30.9
Non-UTIs genitourinary infections	B26.0 B37.3 B37.4 N41.0 N41.2 N41.3 N41.8 N41.9 N43.1 N45.0 N45.9 N48.1 N48.2 N49.0 N49.1 N49.2 N49.8 N49.9 N61.x N70.0 N70.9 N71.0 N71.9 N72.x N73.0 N73.2 N73.3 N73.5 N73.8 N73.9 N75.1 N76.0 N76.2 N76.4 N48.0 N75.8 N76.8 N32.3
Bloodstream infections or sepsis;	A02.1 A20.7 A21.7 A22.7 A26.7 A32.7 A39.1 A39.2 A39.4 A40.0 A40.1 A40.2 A40.3 A40.8 A40.9 A41.0 A41.1 A41.2 A41.3 A41.4 A41.5 A41.8 A41.9 A42.7 A48.3 B00.7 B37.7 R65.1 R65.0 R65.2 U04.9
Abdominal infections	A00.0 A00.1 A00.9 A01.0 A01.1 A01.2 A01.3 A01.4 A02.0 A03.0 A03.1 A03.2 A03.3 A03.8 A03.9 A04.0 A04.1 A04.2 A04.3 A04.4 A04.5 A04.6 A04.7 A04.8 A04.9 A05.0 A05.1 A05.2 A05.3 A05.4 A05.8 A05.9 A06.0 A06.2 A06.3 A06.4 A07.0 A07.1 A07.2 A07.3 A07.8 A07.9 A08.0 A08.1 A08.2 A08.3 A08.4 A08.5 A09.0 A09.9 A21.3 A22.2 A42.1 B05.4 B15.0 B15.9 B16.0 B16.1 B16.2 B16.9 B17.0 B17.1 B17.2 B17.8 B17.9 B19.0 B19.9 B25.1 B25.8 B46.2 K35.0 K35.1 K35.9 K36.x K37.x K57.0 K57.2 K57.4 K57.8 K61.0 K61.1 K61.2 K61.3 K61.4 K63.0 K65.0 K65.9 K75.0 K81.0 K81.8 K81.9 K52.1 K52.9 K57.1 K57.3 K62.8 K63.8 K65.0 K65.8 K83.0
Skin and soft tissue infections	A06.7 A20.1 A22.0 A26.0 A26.8 A26.9 A31.1 A32.0 A36.3 A43.1 A44.1 A46.x A48.0 B00.0 B00.1 B02.9 B07.x B08.8 B09.x B35.0 B35.1 B35.2 B35.3 B35.4 B35.5 B35.6 B35.8 B35.9 B36.0 B36.1 B36.2 B36.3 B36.8 B36.9 B37.2 B38.3 B40.3 B42.1 B43.0 B43.2 B45.2 B46.3 B48.0 L00.x L01.0 L01.1 L02.0 L02.1 L02.2 L02.3 L02.4 L02.8 L02.9 L03.0 L03.1 L03.2 L03.3 L03.8 L03.9 L04.0 L04.1 L04.2 L04.3 L04.8 L04.9 L05.0 L05.9 L08.0 L08.1 L08.8 L08.9 L30.3 L70.2 R02.x L40.1 L40.3 L70.0 L73.2 L84.x L88.x L98.0
Nervous system infections;	A06.6 A20.3 A32.1 A39.0 A83 A85.8 A86.x A87.0 A87.1 A87.2 A87.8 A87.9 A88.8 A89.x B00.3 B00.4 B01.0 B01.1 B02.0 B02.1 B02.2 B05.0 B05.1 B06.0 B26.1 B26.2 B37.5 B38.4

	B43.1 B45.1 B46.1 G00.0 G00.1 G00.2 G00.3 G00.8 G00.9 G04.0 G04.2 G06.0 G06.1 G06.2 G93.7 F05.9 G03.9 G04.8 G04.9 G62.9
Musculoskeletal infections	B33.0 M00.0 M00.1 M00.2 M00.8 M00.9 M46.2 M46.3 M46.5 M60.0 M65.0 M65.1 M71.0 M71.1 M86.0 M86.1 M86.2 M86.8 M86.9 M46.8 M72.8 M94.8
Cardiovascular infections	A39.5 B33.2 B37.6 I01.0 I01.1 I01.2 I01.8 I01.9 I02.0 I02.9 I30.1 I33.0 M05.3 M32.1 I40.0 I410, I411, I412, I430, I39
Device or dialysis related infections	T79.3 T80.2 T81.4 T82.6 T82.7 T83.5 T83.6 T84.5 T84.6 T84.7 T87.4 T88.0 T86.8 T87.1 T87.0 T85.7
Other infections of interest	A02.2 A02.8 A02.9 A06.8 A06.9 A20.0 A20.8 A20.9 A21.0 A21.8 A21.9 A22.8 A22.9 A23.0 A23.1 A23.2 A23.3 A23.8 A23.9 A24.0 A24.1 A24.3 A24.4 A25.0 A25.1 A25.9 A27.0 A27.8 A27.9 A28.0 A28.1 A28.2 A28.8 A28.9 A30.0 A30.1 A30.2 A30.3 A30.4 A30.5 A30.8 A30.9 A31.8 A31.9 A32.8 A32.9 A35.x A39.8 A39.9 A42.2 A42.8 A42.9 A43.8 A43.9 A44.0 A44.8 A44.9 A48.2 A48.4 A48.8 A49.0 A49.1 A49.2 A49.3 A49.8 A49.9 A88.0 A88.1 A90.x A91.x A92.0 A92.1 A92.2 A92.3 A92.4 A93.0 A93.1 A93.2 A93.8 A95.0 A95.1 A95.9 A96.0 A96.1 A96.2 A96.8 A96.9 A98.0 A98.1 A98.2 A98.3 A98.4 A98.5 A98.8 A99.x B00.8 B00.9 B01.8 B01.9 B02.7 B02.8 B03.x B04.x B05.8 B05.9 B06.8 B06.9 B08.0 B08.1 B08.2 B08.3 B08.4 B25.9 B26.8 B26.9 B27.0 B27.1 B27.8 B27.9 B33.3 B33.8 B34.0 B34.1 B34.2 B34.3 B34.4 B34.8 B34.9 B37.0 B37.8 B37.9 B38.7 B38.8 B38.9 B39.0 B39.3 B39.4 B39.5 B39.9 B40.0 B40.7 B40.8 B40.9 B41.7 B41.8 B41.9 B42.7 B42.8 B42.9 B43.8 B43.9 B44.7 B44.8 B44.9 B45.3 B45.7 B45.8 B45.9 B46.4 B46.5 B46.8 B46.9 B47.0 B47.1 B47.9 B48.2 B48.3 B48.4 B48.7 B48.8 B49.x B95.0 B95.1 B95.2 B95.3 B95.4 B95.5 B95.6 B95.7 B95.8 B96.0 B96.1 B96.2 B96.3 B96.4 B96.5 B96.6 B96.7 B96.8 B97.0 B97.1 B97.2 B97.3 B97.4 B97.5 B97.6 B97.7 B97.8 B99.x D73.3 E32.1 I00.x I83.2 K11.2 R17.x D76.2

Supplement Table 2. In-hospital mortality by specific types of infection in 6,283 patients with/without CKD in Guangzhou, China.

In-hospital deaths	Total	Non-CKD	CKD	P-value
Bloodstream infections or sepsis				
Overall, n (%)	39 (18.66)	23 (17.8)	16 (20.0)	0.70
Crude OR		1 (Ref)	1.15 (0.57-2.35)	0.70
Adjusted OR (Model 1)		1 (Ref)	1.02 (0.39-2.66)	0.96
Adjusted OR (Model 2)		1 (Ref)	0.93 (0.35-2.48)	0.89
Pneumonia				
Overall, n (%)	183 (8.25)	111 (7.67)	72 (9.33)	0.17
Crude OR		1 (Ref)	1.23 (0.91-1.69)	0.17
Adjusted OR (Model 1)		1 (Ref)	1.21 (0.87-1.67)	0.23
Adjusted OR (Model 2)		1 (Ref)	1.04 (0.73-1.51)	0.81
Urinary tract infections				
Overall, n (%)	19 (1.5)	7 (0.8)	12 (2.2)	0.02
Crude OR		1 (Ref)	2.83 (1.10-7.24)	0.02
Adjusted OR (Model 1)		1 (Ref)	2.34 (0.67-8.10)	0.18
Adjusted OR (Model 2)		1 (Ref)	2.26 (0.58-8.77)	0.24

Model 1 Adjusted for Age and Sex

Model 2 Adjusted for Age, sex, and Charlson comorbidity index

Supplement Table 3. ICU admission by specific types of infection in 6,283 patients with/without CKD in Guangzhou, China.

ICU admission	Total	Non-CKD	CKD	P-value
Bloodstream infections or sepsis				
Overall, n (%)	82 (39.2)	50 (38.8)	32 (40.0)	0.85
Crude OR		1 (Ref)	1.05 (0.59-1.87)	0.85
Adjusted OR (Model 1)		1 (Ref)	0.70 (0.23-2.16)	0.53
Adjusted OR (Model 2)		1 (Ref)	0.69 (0.22-2.23)	0.62
Pneumonia				
Overall, n (%)	244 (11)	120 (8.3)	124 (16.1)	＜0.001
Crude OR		1 (Ref)	2.12 (1.62-2.77)	＜0.001
Adjusted OR (Model 1)		1 (Ref)	2.51 (1.64-3.01)	＜0.001
Adjusted OR (Model 2)		1 (Ref)	2.53 (1.64-3.88)	＜0.001
Urinary tract infections				
Overall, n (%)	62 (4.2)	26 (2.9)	36 (6.5)	0.001
Crude OR		1 (Ref)	2.34 (1.40-3.92)	＜0.001
Adjusted OR (Model 1)		1 (Ref)	1.96 (0.93-4.15)	0.08
Adjusted OR (Model 2)		1 (Ref)	1.83 (0.82-4.06)	0.14

Model 1 Adjusted for Age and Sex

Model 2 Adjusted for Age, sex and Charlson comorbidity index

Supplement Table 4. In-hospital mortality and ICU admission in patients with/without CKD, excluding readmission in Guangzhou, China.

Odds ratio, OR	Total (n=3,643)	Non-CKD (n=2,631)	CKD (n=1,012)	P-value
**In-hospital death**				
Overall, n (%)	179 (4.9)	107 (4.1)	72 (7.1)	＜0.001*
Crude OR		1 (Ref)	1.81 (1.33-2.46)	＜0.001
Adjusted OR (Model 1)		1 (Ref)	1.37 (0.99-1.90)	0.06
Adjusted OR (Model 2)		1 (Ref)	1.18 (0.84-1.96)	0.33
**Intensive care unit admission**				
Overall, n (%)	262 (7.2)	132 (5.0)	130 (12.9)	＜0.001*
Crude OR		1 (Ref)	2.79 (2.16-3.60)	＜0.001
Adjusted OR (Model 1)		1 (Ref)	1.99 (1.52-2.60)	＜0.001
Adjusted OR (Model 2)		1 (Ref)	1.87 (1.43-2.45)	＜0.001

Model 1 Adjusted for Age and Sex

Model 2 Adjusted for Age, sex and Charlson comorbidity index

* Chi^2 test

Supplement Table 5. The length of hospital stay and associated medical costs in 6,283 patients with/without CKD by different types of infection-related hospitalizations (IRHs) in Guangzhou, China.

	Types of IRHs	Non-CKD	CKD	P-value
**Median days of hospital stay[IQR]**	CAIRHs (n=3,697)	9[6-13]	10[7-14]	＜0.001
	HAIRHs (n=162)	20[14.5-31.5]	28[16-38]	0.27
**Total medical expenses (Median, RMB)**	CAIRHs (n=3,697)	10988.08	12831.11	＜0.001
	HAIRHs (n=162)	36219.94	33785.96	0.46
**Provided medicines (antibiotics excluded)**	CAIRHs (n=3,697)	3971.6	4998.77	＜0.001
	HAIRHs (n=162)	13001.24	12403.98	0.71
**Antibiotics**	CAIRHs (n=3,697)	873.35	1293.17	＜0.001
	HAIRHs (n=162)	2039.21	2495.22	0.23
**Ward-related**	CAIRHs (n=3,697)	1541.3	1976.13	＜0.001
	HAIRHs (n=162)	4439.22	5764.88	0.12
**Investigation-related**	CAIRHs (n=3,697)	2324.98	2659.25	＜0.001
	HAIRHs (n=162)	5141.17	5403.23	0.37
**Non-surgery therapies**	CAIRHs (n=3,697)	1776.32	2400.83	＜0.001
	HAIRHs (n=162)	4800.81	9251.98	0.01
****Surgical therapies, n,(IQR, RMB)**	CAIRHs (n=3,697)	0 (0-32.7)	0(0-66.26)	0.41
	HAIRHs (n=162)	25 (0-1228.4)	62.5(0-1982.5)	0.83

Ward-related costs consider all other cost incurred while in the ward

Investigation-related costs consider pathology, laboratory, imaging and consumable items

Costs of non-surgical therapies include physiotherapy, acupuncture, injection, etc.

Costs of surgical therapies include those of anesthesia and related materials

*Wilcoxon rank test

**Not every patient had a surgery

IQR: interquartile range. CAIRHs: community-acquired infection-related hospitalizations; HAIRHs: health-care associated infection-related hospitalizations

Supplement Table 6. The length of hospital stay and associated medical costs in 6,283 patients with/without CKD by survival group and death group in Guangzhou, China.

	Non-CKD	CKD	Difference in (%)	**P-value	***Difference adjusted by age and sex
Survival group	(n=4,182)	(n=1,820)			
Median days of hospital stay[IQR]	10 [7-14]	11 [8-15]		<0.001	
Total medical cost (Median, RMB)	11788.06	14167.26	20.2	<0.001	1579.9
Provided medicines (antibiotics excluded)	3895.1	5157.25	32.4	<0.001	494.9
Antibiotics	655.02	1080.93	65.0	<0.001	26.6
Ward-related	1496.35	1987.78	32.8	<0.001	310.4
Investigation-related	2531.75	2851.61	12.6	<0.001	413.6
Non-surgical therapies	1905.85	2676.54	40.4	<0.001	394.6
****Surgical therapies, n,(IQR, RMB)	0 (0-246.39)	0 (0-165.03)		0.01	
Death group	(n=166)	(n=115)			
Median days of hospital stay[IQR]	10 [4-18]	9 [4-17]		0.3	
Total medical cost (Median, RMB)	22916.97	22890.22	-0.1	0.94	-4837.8
Provided medicines (antibiotics excluded)	10190.68	8154.86	-20.0	0.21	-3489.0
Antibiotics	1768.37	1363.55	-22.9	0.26	-1507.4
Ward-related	3103.84	3726.84	20.1	0.38	-251.8
Investigation-related	3092.71	4026.05	30.2	0.02	446.0
Non-surgical therapies	3326.52	3992.57	20.0	0.2	-959.7
****Surgical therapies, n,(IQR, RMB)	23.5 [0-350]	180 [0-618]		<0.001	

Data from electronic medical record database in Guangdong provincial hospital of Chinese medicine.

Ward-related costs consider all other cost incurred while in the ward

Investigation-related costs consider pathology, laboratory, imaging and consumable items

The cost of non-surgical therapies includes physiotherapy, acupuncture, injection, etc.

Cost of surgical therapies include those of anesthesia and related materials

Difference=[(Cost in CKD - Cost in Non-CKD)/Cost in Non-CKD]×100%

All medical costs were adjusted for inflation rate based on cost in 2012; Inflation rate equals to 2.62% for 2013, 1.92% for 2014 and 1.44% for 2015 from http://www.inflation.eu/inflation-rates/china/historic-inflation/cpi-inflation-china.aspx

**Wilcoxon rank test

***General linear model adjusted for age and sex

****Not every patient had a surgery

Abbreviations: IQR: interquartile range
